# Static and dynamic brain functional connectivity patterns in patients with unilateral moderate-to-severe asymptomatic carotid stenosis

**DOI:** 10.3389/fnagi.2024.1497874

**Published:** 2025-01-15

**Authors:** Junjun Wang, Linfeng Song, Binlin Tian, Li Yang, Xiaoyu Gu, Xu Chen, Lei Gao, Lin Jiang

**Affiliations:** ^1^Department of Radiology, The Third Affiliated Hospital of Zunyi Medical University (The First People's Hospital of Zunyi), Zunyi, Guizhou, China; ^2^Department of Radiology, Zhongnan Hospital of Wuhan University, Wuhan, China

**Keywords:** carotid stenosis, fMRI, dynamic functional connectivity, functional connectivity, dynamic independent component analysis

## Abstract

**Background and purpose:**

Asymptomatic carotid stenosis (ACS) is an independent risk factor for ischemic stroke and vascular cognitive impairment, affecting cognitive function across multiple domains. This study aimed to explore differences in static and dynamic intrinsic functional connectivity and temporal dynamics between patients with ACS and those without carotid stenosis.

**Methods:**

We recruited 30 patients with unilateral moderate-to-severe (stenosis ≥ 50%) ACS and 30 demographically-matched healthy controls. All participants underwent neuropsychological testing and 3.0T brain MRI scans. Resting-state functional MRI (rs-fMRI) was used to calculate both static and dynamic functional connectivity. Dynamic independent component analysis (dICA) was employed to extract independent circuits/networks and to detect time-frequency modulation at the circuit level. Further imaging-behavior associations identified static and dynamic functional connectivity patterns that reflect cognitive decline.

**Results:**

ACS patients showed altered functional connectivity in multiple brain regions and networks compared to controls. Increased connectivity was observed in the inferior parietal lobule, frontal lobe, and temporal lobe. dICA further revealed changes in the temporal frequency of connectivity in the salience network. Significant differences in the temporal variability of connectivity were found in the fronto-parietal network, dorsal attention network, sensory-motor network, language network, and visual network. The temporal parameters of these brain networks were also related to overall cognition and memory.

**Conclusions:**

These results suggest that ACS involves not only changes in the static large-scale brain network connectivity but also dynamic temporal variations, which parallel overall cognition and memory recall.

## 1 Introduction

Clinically, asymptomatic carotid stenosis (ACS) is defined by the narrowing of the extracranial internal carotid artery without recent stroke or transient ischemic attacks (TIA) (Coutts et al., 2015; Inzitari et al., 2000). Despite the absence of overt neurological events, ACS is increasingly recognized for its association with cognitive complaints, particularly in the domains of memory and executive function (Hu et al., 2023; Lazar et al., 2021). Even mild forms of ACS can significantly impact daily living activities (Ghaznawi et al., 2023). However, the neural mechanisms underlying the cognitive impairments observed in ACS remain poorly understood.

Recent research has leveraged large-scale brain networks to understand both normal brain function and neurological disorders (Wang et al., 2021). Functional connectivity (FC) magnetic resonance imaging (MRI), which measures the statistical dependencies between low-frequency blood oxygen level-dependent (BOLD) signals across brain regions, has enabled the identification of distinct intrinsic large-scale brain networks (Biswal et al., 1995). These include primary networks such as the visual networks (VIN), auditory, and somatosensory/motor networks (SMN), as well as higher-order networks like the default mode network (DMN), salience network (SN), language network (LN), fronto-parietal network (FPN), and dorsal attention network (DAN) for a review see (Raichle, 2011). The spatiotemporal dynamics of these networks are considered essential for normal brain function and cognition (Omidvarnia et al., 2021). Moreover, several major neurodegenerative diseases (Filippi et al., 2019), including Alzheimer's disease (AD; Núñez et al., 2021), traumatic brain injury (van der Horn et al., 2020), and Parkinson's disease (Dirkx et al., 2023), have been shown to involve alterations in both the spatial organization and temporal dynamics of these brain networks.

Using static FC (sFC), previous studies have identified altered synchronization of spontaneous brain activity both within and between hemispheres in ACS patients, particularly in SM, DAN, SN, and DAN near the lateral sulcus (Gao et al., 2019). Huang et al. (2020) found reduced activity in the left occipital gyrus of ACS patients. Seed-based analysis further revealed decreased FC between the left occipital gyrus and the FPN. Fan et al. (2024) reported hemispheric asymmetry in intrinsic activity in ACS patients, with structural and functional asymmetry decoupled. They also found that declines in simple cognitive functions, such as delayed memory recall and sensory-motor processing, were closely related to weakened regional FC. These findings suggest that static resting-state brain network connectivity is altered in multiple large-scale networks both within and between hemispheres in ACS patients, but how the temporal dynamics of these networks change remains unclear.

Dynamic FC (dFC) provides a means to assess brain network dynamics by characterizing variations in temporal fluctuations along the time axis (Preti et al., 2017). In contrast to sFC, which reflects the averaged correlations of time series, dFC captures time-dependent changes, thus offering a more nuanced view of the temporal variability and coupling dynamics within large-scale brain networks (Fan et al., 2021). In recent years, dFC has gained considerable attention as a research tool, demonstrating its ability to detect subtle alterations in brain network dynamics in various neurological conditions, including AD (Zhao C. et al., 2022). Given that ACS represents a chronic hemodynamic disruption associated with brain atrophy, an increased microinfarct burden, and cognitive decline (Wang et al., 2017; Paraskevas et al., 2023), the application of dFC may provide valuable insights into the altered dynamics of spontaneous brain activity at both regional and system levels. This approach holds potential for advancing our understanding of the pathological mechanisms underlying cognitive impairment in ACS.

In this study, we aimed to investigate changes in the temporal variability and frequency of large-scale brain networks in patients with ACS using dFC. We hypothesized that ACS patients exhibit altered temporal variability in brain regions around the lateral sulcus, involving multiple brain networks, and that these changes are associated with deficits in memory and executive function.

## 2 Materials and methods

### 2.1 Participants

We collected data from 36 ACS patients and 33 healthy controls (HC) who were matched for comorbidities (i.e., hypertension, diabetes) and demographics (i.e., age, gender, and education). The ACS patients, aged between 50 and 80 years, right-handed, were recruited between January and December 2023. The inclusion criteria for ACS patients were as follows: (a) moderate to severe (50%−99%) unilateral internal carotid artery stenosis, as per North American Symptomatic Carotid Endarterectomy Trial criteria (Moneta et al., 1993; Staikov et al., 2002); (b) no history of stroke, TIA, or other lesions on conventional MRI; (c) no significant cognitive impairment or dementia, with a Mini-Mental State Examination (MMSE) score ≥24 (Tombaugh and McIntyre, 1992) and Montreal Cognitive Assessment Beijing Version (MoCA) score ≥26 (Nasreddine et al., 2005); (d) functional independence (Modified Rankin Scale ≤ 1) (Banks and Marotta, 2007); and (e) a minimum of 6 years of education. Exclusion criteria included: (a) bilateral internal carotid artery stenosis ≥50%; (b) posterior circulation diseases; (c) severe systemic or neuropsychiatric disorders; (d) contraindications for MRI (e.g., metal implants, cardiac pacemaker, claustrophobia, or inability to cooperate for scanning); and (e) illiteracy. This study serves as the dataset for one of the centers in a multi-center research project and has been approved by the Medical Ethics Committee of the Third Affiliated Hospital of Zunyi Medical University, and all participants gave written informed consent. Detailed demographics and clinical measures are listed in Table 1.

**Table 1:** Participant characteristics and clinical information.

	**HC (*n =* 30)**	**ACS (*n =* 30)**	***χ2/t/Z***	***p***
Gender (male/female)	20/10	19/11	0.109^#^	0.742
Age (yrs.), mean ± SD	62 ± 6.4	64 ± 7.0	1.128^##^	0.264
Education (yrs.)	9 (9, 12)	9 (9, 12)	−0.695^#^	0.487
Hypertension, *n* (%)	13 (43.3%)	17 (56.7%)	0.295^#^	0.587
Diabetes, *n* (%)	13 (43.3%)	14 (46.7%)	0.645^#^	0.422
Drink, *n* (%)	15 (50%)	12 (40.0%)	0.783^#^	0.376
Smoking, *n* (%)	12 (40.0%)	13 (43.3%)	0.380^#^	0.846
Stenosis side	N/A	15L/15R	/	/
Stenosis rate	N/A	25/5	/	/
MMSE	28.00 (27.00, 29.00)	27.00 (27.00, 28.25)	−2.215^##^	0.027^*^
MoCA	27.00 (26.75, 28.00)	26.00 (25.00, 26.25)	−3.080^##^	0.002^*^
Word fluency	37.00(36.00, 38.25)	34.00(31.75, 36.00)	−4.975^###^	< 0.001^**^
DST	29.50 (28.00, 32.25)	27.00 (26.00 32.25)	−5.501^###^	< 0.001^*^
Backwards span	6.00 (5.00, 7.00)	6.00 (6.00, 6.00)	−1.483^###^	0.138
Forwards span	7.00 (7.00, 8.00)	7.00 (7.00, 7.25)	−1.622^###^	0.105
Immediate recall, mean±SD	33.70 ± 1.91	31.13 ± 2.08	4.973^##^	< 0.001^*^
Delayed recall	6.00 (5.00, 7.00)	5.00 (4.25, 5.25)	−2.920^###^	0.04^*^

Two-sample *t* test was used for data conforming to normal distribution and homogeneity of variance, and the data were expressed as mean ± SD. The Mann-Whitney U test was used for data conforming to normal distribution but with uneven variance or non-conforming to normal distribution, and the data are expressed as medians (first and third quartiles). The categorical variables use a chi-square test, and the data are expressed as *n* (%). Among the 30 ACS patients, 25 had moderate unilateral carotid stenosis, and 5 had severe unilateral carotid stenosis. ACS, asymptomatic carotid stenosis group; HC, healthy control group; MMSE, Mini-Mental State Examination; MoCA, Montreal Cognitive Assessment; L, left; R, right. # chi-square Test; ## Two-sample *t* Test; ### Mann- Whitney U Test. ^*^p < 0.05, ^**^p < 0.001, respectively.

### 2.2 Neurobehavioral assessment

All participants completed a comprehensive cognitive test before the MRI scan, neurobehavioral assessment was mainly applied with the MMSE, MoCA, Digit Span Test (DST; Strauss et al., 2006) and Rey Auditory Verbal Learning (RAVLT; Schmidt, 1996) for, which are performed prior to the MRI scan. These tests measure cognitive domains including (1) global cognition: MMSE and MoCA; (2) information processing speed: DST, using the Digit Span Forwards Test and the Digit Span Backwards Test; and (3) memory and language learning ability: RAVLT.

### 2.3 MRI data acquisition

Imaging was performed using a 3.0 T Siemens MAGNETOM Vida MRI scanner with a 32-channel head coil. The data sequences included (i) The resting-state blood oxygenation level dependent (BOLD) signal was performed using a single-shot echo-planar sequence with the following parameters: 33 axial slices, 3.8 mm thick, gap 1 mm, Repetition Time (TR) = 2,000 ms, Echo Time (TE) = 30 ms, matrix size = 256 × 256 mm^2^, field of view (FOV) = 256 × 256 mm^2^, duration 8 min; (ii) high-resolution 3D-T_1_ weighted structural images obtained using the magnetization-prepared rapid gradient-echo (MPRAGE) sequence for: TR/TE = 2,300/2.98 ms, voxel size = 3.75 × 3.75 × 3.8 mm^3^, flip angle (FA) = 12°, gap 0 mm, sagittal slices, matrix size = 256 × 256 mm^2^, FOV = 256 × 256 mm^2^); (iii) T_2_WI-fluid-attenuated inversion recovery (T_2_-FLAIR) images: [TR/TE/Inversion Time (TI) = 6,000/395/2,200 ms, FA = 90°, voxel size = 0.5 × 0.5 × 1.0 mm^3^, 160 axial slices, FOV = 230 × 230 mm^2^]. Participants' heads were lightly restrained using soft pads to prevent head movement. Subjects were instructed to rest quietly with their eyes close and to remain rest during the scan.

### 2.4 rs-fMRI data processing

The CONN toolbox (CONN; https://www.nitrc.org/projects/conn, version 21. a) was used based on the Statistical Parameter Mapping (SPM12) program (http://www.fil.ion.ucl.ac.uk/spm, version 7771). For each participant, a standard preprocessing procedure was used based on recent studies (Luppi et al., 2023, 2024), including (1) discarding of the first ten volumes to ensure steady-state longitudinal magnetization; (2) head motion realignment: first, we examined each participant during the scan images, and eliminate any translation for more than 2 mm head movement during the period of TR exists; second, rigid body registration for inter-frame head motion; thirdly, compensation of systematic slice-dependent time shifts by phase shift in the Fourier domain; (3) functional slice timing correction: to ensure that all voxels within the same volume had been acquired simultaneously, the slice time was corrected based on slice order, and the middle slice was chosen as the slice to reference; (4) Artifact Detection Tools (ART; https://www.nitrc.org/projects/artifact_detect): outlier scans for scrubbing was performed to remove the aberrant time points (Carruzzo et al., 2022); (5) co-alignment of the T_1_ images; and (6) spatial normalization to the standard Montreal Neurological Institute (MNI) space (resampled to 3 × 3 × 3 mm^3^ isotropic spatial resolution); (7) segmentation of functional and structural data into gray matter, white matter, and cerebrospinal fluid tissues; and (8) spatial smoothing at half-maximum using a Gaussian kernel with a full width of 8 mm. The default settings of the CONN Toolbox, which contains 20 separate components, were used. At each step, the processed functional and anatomical images were carefully visually inspected.

To further reduce cardiac and motion artifacts, an anatomical component-based noise correction method (aCompCor; Muschelli et al., 2014) was applied to remove artifacts from the functional data implemented in the CONN toolbox. Specifically, the aCompCor method was used to remove several potential confounding effects: five potential noise components in the white matter and cerebrospinal fluid signals; estimated motion parameters (three translational and three rotational parameters and their associated first-order derivatives); data that were identified by ART; and that predominantly affect scanning conditions. Finally, linear detrending was applied to minimize low-frequency drift effects and high-frequency noise by band-pass filtering at frequencies of 0.008–0.09 Hz.

Structural T_1_ images were skull-stripped, segmented, and normalized to the MNI template as well yielding normalized structural volumes. Differences in FC between the ACS and HC groups were determined at two levels while controlling for the effects of age and gender using: (i) ICA and (ii) dICA.

### 2.5 Dynamic independent component analysis

The ICA enables the disaggregation and organization of voxels associated with the time course of BOLD signals in specific brain regions into spatially independent component (IC), it's beneficial to the next step of the analysis. Therefore, after data preprocessing, the group-ICA method was selected in the CONN toolbox, and both groups of subjects were entered into the ICA analysis to assess the number of components in the dataset for all subjects to determine the major brain networks of the two groups, and the results show the resting state networks between the groups. The results were identified by visual inspection of the functional networks (as well as noise components) and confirmed by comparison with functional networks reported in previous studies. In addition, we confirmed the validity of the identified networks by estimating the correlation between each group-level spatial map and the CONN default template network.

dICA analyses were performed by iterative double regression on the BOLD time series data for all participant connections to examine the temporal characteristics of brain FC, followed by ICA analyses (controlling for confounding variables) and generalized psychophysiological interactions (gPPI) back-projection. Since differences in the resting state component between groups may be caused by changes in connectivity between specific regions in that component, the method essentially consists of a gPPI interaction term between the component time series as a psychological factor and the region of interest (ROI) BOLD time series as a physiological factor. This technique identifies clusters of connections that exhibit similar patterns of temporal functional change in FC over time. The number of factors was set to 20, and the smoothing kernel was set to 30 s per the default setting of CONN. The calculation of dFC was performed using the 32 brain network nodes provided by the CONN toolbox. Specifically, the CONN toolbox divides the human brain into 8 large-scale brain networks by default, which include the SN, VIN, DAN, LN, DMN, SM, FPN, and the Cerebellar Network (CN). These 8 large-scale networks are then mapped onto 32 brain regions, which are used to compute dFC. The correction for dFC was performed at the connection level with a threshold of *p* < 0.05 (two-tailed), and at the cluster level with a *p*-value threshold of < 0.05, corrected for false discovery rate (FDR) using a multivariable omnibus test.

### 2.6 Statistical analysis

A total of 69 participants met the study's inclusion and exclusion criteria and underwent rs-fMRI scanning. However, 6 ACS patients and 3 HC participants were excluded due to motion artifacts. The final cohort consisted of 60 participants, including 30 ACS patients and 30 HC participants individuals (Table 1). Among the ACS patients, 25 had moderate stenosis (approximately 50%−69%) and 5 had severe stenosis (70%−99%). All clinical, cognitive and demographic variables were analyzed using SPSS 29.0 (IBM Crop., Armonk, NY, USA, Version 29.0) for between-groups statistics at a significance level of *p* < 0.05. For age, cognitive test score, which followed a normal distribution and had homogeneous variances, an independent two-sample *t-*test was used. For variables that followed a normal distribution but did not meet the assumption of homogeneity of variances, or those that did not follow a normal distribution, the Mann-Whitney U test was applied for inter-group comparisons. For categorical variables, including gender, diabetes, education, hyperlipidemia, and smoking status, *Chi*-square tests were used to determine group differences, with statistical significance set at *p* < 0.05.

For imaging data, all statistical group-level comparisons were implemented in the CONN toolbox. In ICA comparisons, cluster-level FDR corrected threshold was set at *p* < 0.05 based on Gaussian random field theory. In ICA comparisons, the cluster-level FDR correction threshold was set to *p* < 0.05 based on Gaussian random field theory. In dICA comparisons, cluster-based inference was based on a cluster-level *p*- FDR correction threshold of *p* < 0.05 and tested using multivariate pattern analysis (MVPA) synthesis.

To understand how the observed differences in static and dynamic resting-state brain activity relate to cognitive performance, we performed *Pearson* correlations at both the voxel-level and the connection-level to explore brain-cognition associations in clusters or connections that exhibited group differences.

## 3 Results

### 3.1 Characteristics of participants

As compared to HC, ACS patients had worse memory (immediate recall, *p* < 0.001; delayed recall, *p* < 0.05), executive functioning (DST, *p* < 0.001) and word fluency (*p* < 0.001) than controls (Table 1).

### 3.2 ICA results

**Figure 1:**
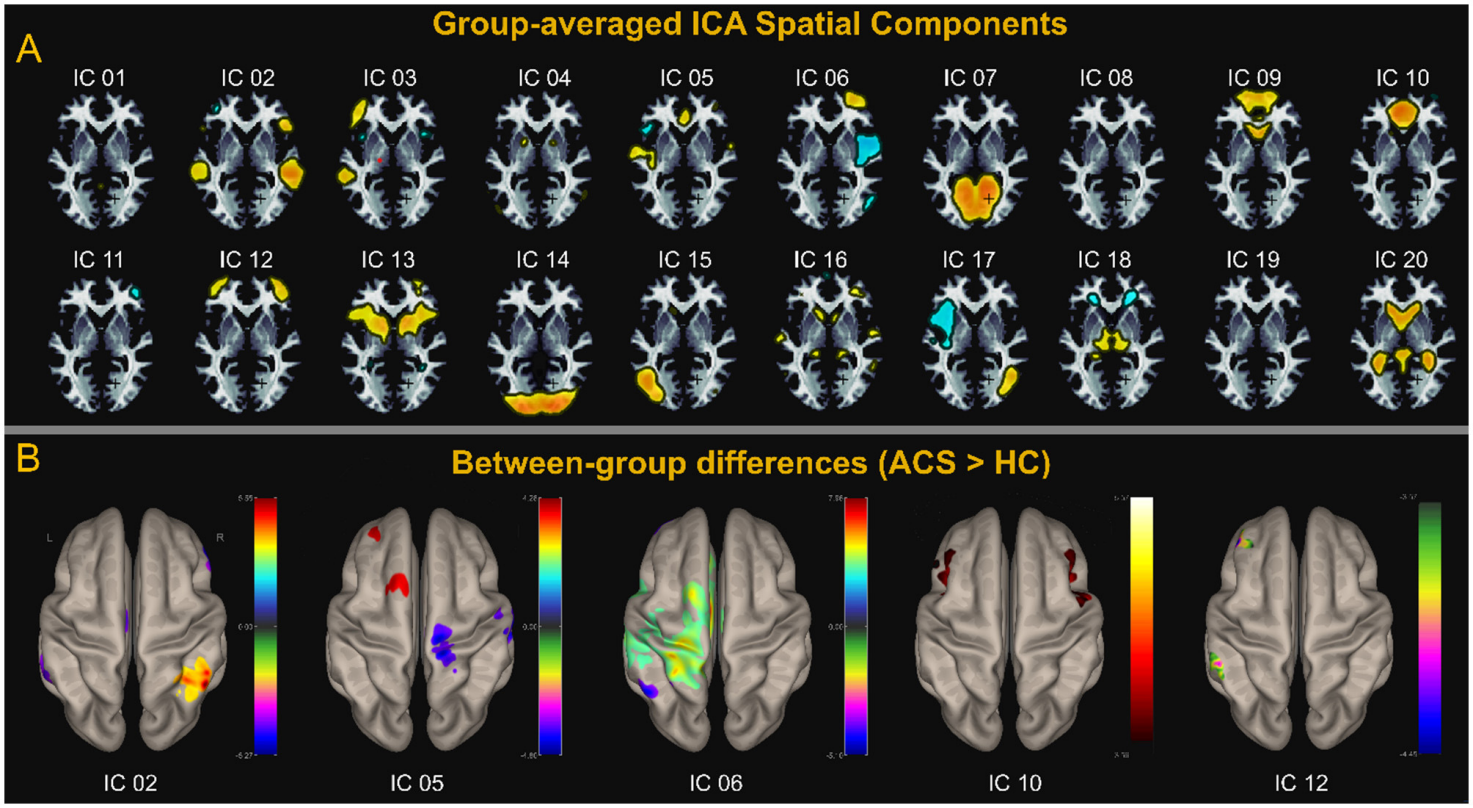
ICA analysis. **(A)** Shows the spatial distribution of the 20 components estimated using group-level averaged ICA. **(B)** Shows Components LN (IC_02), SM (IC_05), and FPN_L (IC_06 and IC_12) correspond to the LN, SM, FPN, and SN, respectively. **(B)** Displays the components with group differences and the corresponding brain anatomy. Except for SN (IC_10), which shows significantly higher spontaneous brain activity in the ACS group compared to the control group, the other four networks—LN (IC_02), SM (IC_05), FPN_L (IC_06 and IC_12) —exhibit regions with higher spontaneous brain activity in the HC group compared to the ACS. The values on the adjacent colorbar reflect the magnitude of the statistical *t*-values. A negative *t*-value (represented by cool colors on the jet colormap) indicates that the ACS group has lower activity than the control group, while a positive *t*-value (represented by warm colors on the jet colormap) indicates that the ACS group has higher activity than the control group.

We first report the spatial differences of ICA. We estimated 20 ICs and matched these components with large-scale brain network templates, adapting the best-fitting brain network for each component, as shown in Figure 1A. We performed voxel-wise group comparisons for components that reflect brain networks, within the corresponding brain network component masks. We first report the spatial differences of ICA. We estimated 20 ICs and matched these components with large-scale brain network templates, adapting the best-fitting brain network for each component, as shown in Figure 1A. We performed voxel-wise group comparisons for components that reflect brain networks, within the corresponding brain network component masks. ACS brain networks - LN (IC_02), SM (IC_05), and FPN_L (IC_06 and IC_12)-showed significant group differences, specifically, sFC was enhanced in the SN, while it was reduced in the FPN-L, SM, and LN, as shown in Figure 1B. These results were corrected for voxel-level *p* < 0.001 and cluster-level *p* < 0.05 using FDR cluster correction.

### 3.3 dICA results

**Figure 2:**
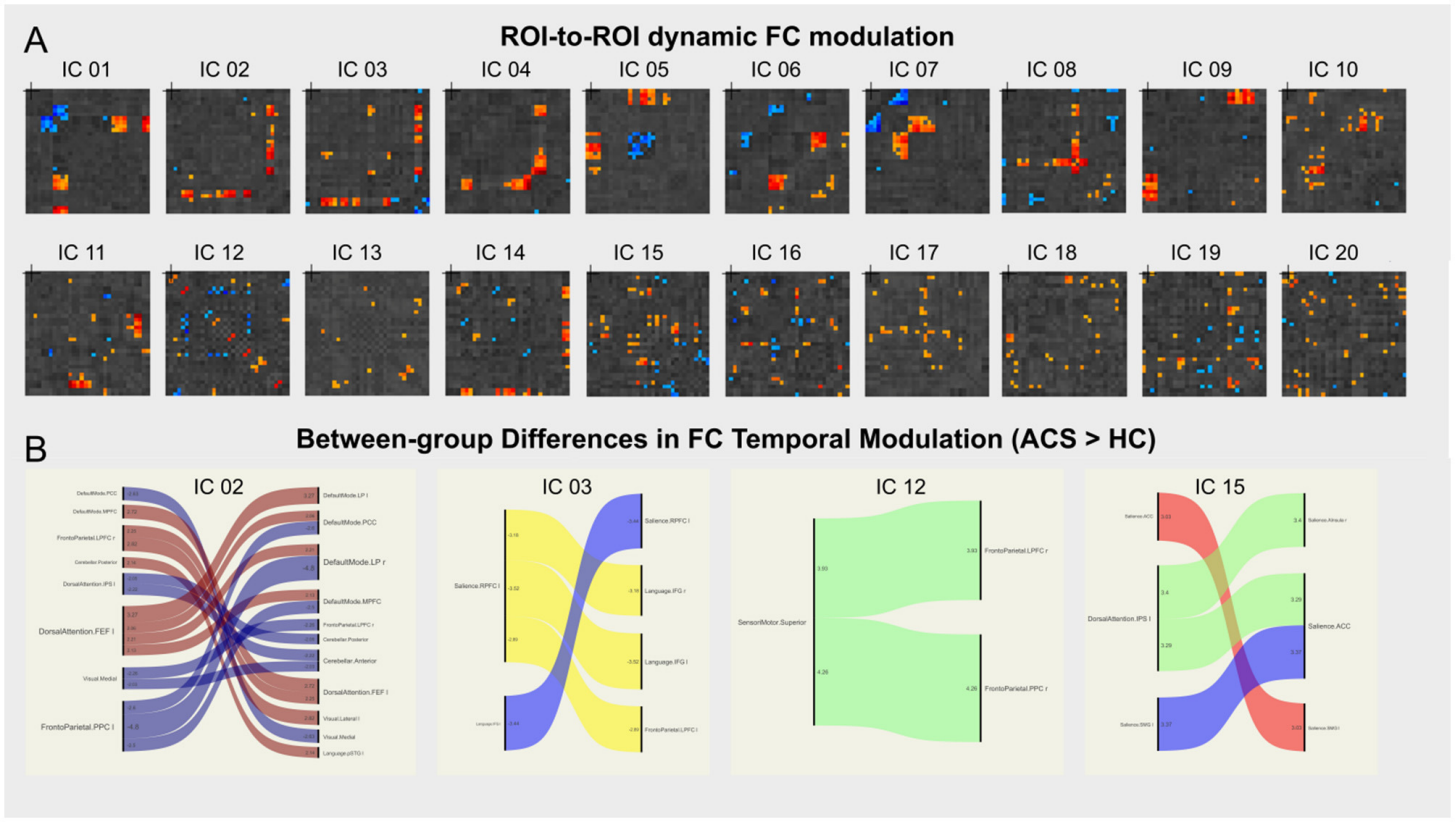
dICA analysis. **(A)** Shows the ROI-to-ROI connectivity matrix for the 20 ICs spatial components, with the factor loadings for each connection color-coded by circuit. **(B)** Shows the circuit connections with significant group differences, for example, brain networks—LN (IC_02), FPN_L (IC_12), VIN (IC_15), and IC_03 shows significantly higher FC temporal modulation in the ACS group compared to the control group. A negative *t-*value indicates lower connectivity in the ACS group compared to the control group, while a positive *t*-value indicates higher connectivity in the ACS group.

**Figure 3:**
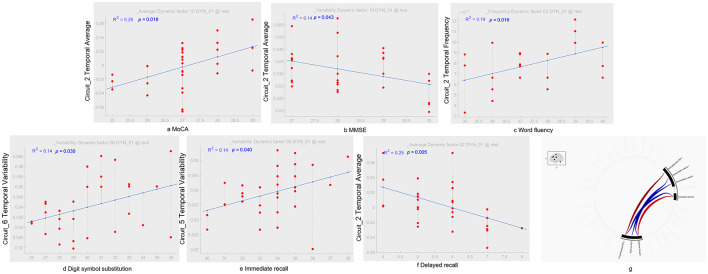
dICA temporal components and their correlation with cognitive tests. In terms of temporal average characteristics, IC_02 in the ACS group was positively correlated with MoCA [*r*^2^ = 0.25, *p* = 0.018; as shown in **(A)**], which was positively correlated with MMSE [*r*^2^ = 0.14, *p* = 0.043; as shown in **(B)**], and delayed recall [*r*^2^ = 0.25, *p* = 0.005; as shown in **(F)**]. Regarding time-frequency characteristics, IC_02 in the ACS group was positively correlated with language fluency [*r*^2^ = 0.19, *p* = 0.016; as shown in **(C)**]. In terms of temporal-variability characteristics, IC_06 in the ACS group was positively correlated with digit symbol substitution [*r*^2^ = 0.14, *p* = 0.039; as shown in **(D)**], while IC_05 was positively correlated with immediate recall [*r*^2^ = 0.14, *p* = 0.040; as shown in **(E)**]. Using GLM, regions with dFC differences between the ACS and HC groups were identified, with cluster-based inference using a *p*-FDR corrected threshold of *p* < 0.05, and MVPA was applied for comprehensive testing [as shown in **(G)**]. GLM, generalize linear model; MVPA, multivariate pattern analysis.

dICA analyzes group-ICA and the properties of the dICA (i.e., kurtosis, skewness, temporal variability, and frequency), creating a z-score based threshold that includes factors associated with each individual connection. These factors are color-coded in the ROI-to-ROI connection matrix. Next, the connection time series is reconstructed, and a connection matrix is established to show the change in ROI-to-ROI connection values over time (as shown in Figure 2A). These dynamic changes in FC are then analyzed by applying thresholds based on both connection-level and seed or network-level factors. Non-parametric analysis, along with other display and analysis options, is performed to generate a correlation connection graph (as shown in Figure 3G).

Further analysis using dICA revealed significant differences in the temporal dimensions (i.e., kurtosis, variability, skewness, and frequency) of 20 ICs in ACS, as shown in Table 2. These differences were then mapped to eight brain networks, and dFC characteristics were analyzed for all participants, as detailed in Table 3. The results indicated significant higher FC temporal modulation in patterns in ACS, particularly in the brain networks - LN (IC_02), FPN_L (IC_12), VIN (IC_15), and IC_03 (as shown in Figure 2B).

**Table 2:** Properties of the estimated circuits/ICs.

**Circuits**	**Kurtosis**	**Skewness**	**Variability**	**Frequency**	**Networks**
Circuit_01	7.4871	0.81966	0.04208	0.0076419	CN
Circuit_02	7.4992	1.5528	0.040622	0.0076673	LN
Circuit_03	6.1631	0.93403	0.036082	0.0080227	NA
Circuit_04	7.5947	1.7438	0.041741	0.0080735	CN
Circuit_05	6.6679	−0.092573	0.044547	0.0083528	SM
Circuit_06	6.0216	0.82233	0.041496	0.0079466	FPN_L
Circuit_07	6.3938	0.23157	0.043369	0.0079212	VIN
Circuit_08	6.2983	1.07	0.035887	0.0086067	NA
Circuit_09	6.9855	1.5486	0.03759	0.0086828	DMN
Circuit_10	4.6128	0.62269	0.039701	0.0072865	SN
Circuit_11	5.9941	0.75296	0.038741	0.0082766	DMN
Circuit_12	4.5638	0.26007	0.02683	0.0086321	FPN_L
Circuit_13	2.4054	0.080623	0.043079	0.0075404	SN
Circuit_14	4.0022	0.62158	0.040806	0.0081497	VIN
Circuit_15	3.6254	0.3115	0.033927	0.0076419	VIN
Circuit_16	3.5504	0.042327	0.031083	0.0084797	DAN
Circuit_17	3.6126	0.24249	0.032835	0.0081751	DAN
Circuit_18	3.4553	−0.042262	0.035529	0.007972	NA
Circuit_19	2.82	0.2469	0.036745	0.0079466	SM
Circuit_20	2.8643	0.25284	0.039957	0.0074896	CN

This table lists the properties of the 20 estimated circuits/ICs, including kurtosis, skewness, variability, frequency, and brain networks. Components IC_01, IC_04 and IC_20 correspond to the CN; IC_02 correspond to the LN; IC_06, IC_12 correspond to the FPN_L; IC_05, IC_29 correspond to the SM; IC_07, IC_14 and IC_15 correspond to the VIN; IC_09 correspond to the DMN; IC_11 correspond to the DMN; IC_10 correspond to the SN, IC_10 correspond to the SN; IC_16,IC_17 correspond to the DAN, respectively. IC, independent component; LN, language network; FPN, fronto-parietal network; SM, sensory motor; SN, salience network; DAN, dorsal attention network; VIN, visual network; DMN, default mode network; CN, Cerebellar Network; NA, null-able values; L, left hemisphere.

**Table 3:** Comparison of network differences using dICA.

	**X**	**Y**	**Z**	**Size**	***p*-unc**	***p*-FDR**	**Region**	**Direction**
**LN**								
	−54	−12	−2	281	0.000004	0.000006	Temporal_Sup_L	POS
	−46	−54	12	270	0.000006	0.000006	Temporal_Mid_L	NEG
IC_02	+46	−50	+54	660	< 0.000001	0.000009	Parietal_Inf_R	NEG
	−56	−44	+34	381	0.000008	0.000390	SupraMarginal_L	NEG
	−52	−22	−02	377	0.000002	0.000390	Temporal_Mid_L	NEG
	+56	+30	+04	343	< 0.000001	0.000567	Frontal_Inf_Tri_R	NEG
	−02	−16	+46	140	0.000028	0.043909	Cingulate_Mid_L	NEG
**FPN**								
6	34	14	6	119	0.000033	0.000037	Insula_R	POS
	22	30	50	133	0.000051	0.000051	Frontal_Sup_2_R	NEG
	0	−2	42	156	0.000008	0.000012	Cingulate_Mid_L	POS
	−38	−72	−34	165	0.000024	0.000032	Cerebellum_Crus1_L	NEG
	−56	−40	30	176	0.000006	0.000012	SupraMarginal_L	POS
	48	0	56	204	0.000001	0.000003	Frontal_Mid_2_R	POS
	48	22	−10	210	0.000001	0.000003	Frontal_Inf_Orb_2_R	NEG
	48	−60	38	346	0.000033	0.000037	Angular_R	POS
	64	−38	36	413	0.000006	0.000012	SupraMarginal_R	NEG
	36	−62	−42	553	0.000001	0.000003	Cerebellum_Crus1_R	NEG
	64	−20	−24	798	< 0.000001	0.000001	Temporal_Inf_R	POS
	10	16	34	981	< 0.000001	< 0.000001	Cingulate_Mid_R	NEG
IC_06	−08	−06	+42	9,161	< 0.000001	< 0.000001	Cingulate_Mid_L	NEG
	+32	−32	+02	401	0.000001	0.000432	/	NEG
	−42	−56	+54	235	0.000014	0.007411	Parietal_Inf_L	NEG
	−40	+56	−06	198	0.000001	0.012610	Frontal_Mid_2_L	NEG
	+30	−38	+22	169	0.000014	0.019882	/	NEG
	−08	+24	+30	154	0.000005	0.023892	/	NEG
11	−36	58	0	656	< 0.000001	< 0.000001	Frontal_Sup_2_L	NEG
	40	−8	−30	264	< 0.000001	< 0.000001	Fusiform_R	POS
	−48	−56	54	137	0.000060	0.000060	Parietal_Inf_L	NEG
IC_12	−60	−38	+50	193	0.000004	0.017958	/	NEG
	−44	+46	+24	182	0.000007	0.017958	Frontal_Mid_2_L	NEG
**DAN**								
	−28	−36	26	610	< 0.000001	< 0.000001	/	POS
	2	−36	0	208	0.000002	0.000005	/	POS
	−26	−6	62	207	0.000004	0.000007	Frontal_Sup_2_L	NEG
	26	−4	46	170	0.000013	0.000019	/	NEG
	34	−54	64	408	0.000026	0.000031	Parietal_Sup_R	NEG
	−38	−36	42	366	0.000048	0.000048	Postcentral_L	NEG
**VIN**								
	−36	−64	−6	142	0.000002	0.000005	/	NEG
	42	−74	48	236	0.000023	0.000023	Angular_R	POS
**SM**								
	−46	−12	32	503	0.000002	0.000005	Postcentral_L	NEG
	58	−4	22	279	0.000025	0.000025	Postcentral_R	NEG
IC_05	+44	−22	+16	918	0.000003	< 0.000001	Rolandic_Oper_R	NEG
	+14	−28	+80	453	0.000007	0.000109	Precentral_R	NEG
	−08	+16	+44	259	0.000049	0.003382	Supp_Motor_Area_L	NEG
	−44	+26	+00	160	0.000003	0.026208	Frontal_Inf_Tri_L	NEG
	−28	+38	+16	132	0.000003	0.044048	Frontal_Mid_2_L	NEG
**DMN**								
	22	−48	12	543	0.000001	0.000003	Precuneus_R	POS
	−26	8	−44	129	0.000002	0.000003	Fusiform_L	POS
	12	10	48	141	0.000024	0.000024	Supp_Motor_Area_R	NEG
**SN**								
	−20	−66	−38	444	< 0.000001	0.000001	Cerebellum_8_L	POS
	38	−12	48	165	0.000011	0.000016	Precentral_R	POS
	10	42	36	176	0.000031	0.000031	Frontal_Sup_Medial_R	POS
IC_10	−38	+14	+34	799	< 0.000001	< 0.000001	Frontal_Mid_2_L	POS
	+56	+10	+38	710	0.000013	0.000001	Precentral_R	POS

Comparison of connectivity differences in FC networks base on dICA. X, Y, and Z represent peak-voxel location within each cluster in the MNI space; Size represents the size of the cluster; *p*-FWE represents cluster-size *p*-value corrected for family wise error; *p*-FDR represents cluster-size *p*-value corrected for false discovery rate. NEG indicates that the connectivity of the ACS group is lower than that of the control group, while POS indicates that the connectivity of the ACS group is higher than that of the control group. MNI, Montreal neurological institute; NEG, negative; POS, positive; R, right hemisphere.

Specifically, dynamic connectivity at the network level showed significant differences between the ACS and HC groups (as shown in Table 3). Compared to the HC group, the ACS group exhibited statistically significant differences in connectivity patterns across seven major brain networks (FPN, DMN, SM, SN, DAN, VIN, and LN). Specifically, the ACS group showed significantly weakened FC in the bilateral postcentral gyrus (PoCG), dorsolateral prefrontal cortex (dlPFC), cerebellar Crus1, left superior temporal gyrus (STG.L), left middle temporal gyrus (MTG.L), right superior parietal gyrus (SPG.R), right middle frontal gyrus (MFG.R), right fusiform gyrus (FFG.R), and right inferior temporal gyrus (ITG.R). In contrast, FC was significantly enhanced in the bilateral superior frontal gyrus (SFG), middle cingulate gyrus (MCG), supramarginal gyrus (SMG), left cerebellum_8, left fusiform gyrus (FFG.L), IPL.L, right precentral gyrus (PreCG.R), right middle frontal gyrus (MFG.R), IPL.R, right inferior orbital gyrus (ORBinf.R), right insula (INS.R), right precuneus (PCUN.R), right supplementary motor area (SMA.R), and left angular gyrus (ANG.L) (as shown in Table 3).

Furthermore, our results revealed significant group-level differences in the time-space characteristics between ACS and HC groups in the SN, FPN, DAN, SM, and VIN networks (as shown in Table 4). Compared to the HC group, the ACS group exhibited significant differences in temporal-frequency characteristics in the SN (*p* < 0.05), while significant differences in temporal-variability characteristics were observed in the FPN, DAN, VIN, and SM networks (*p* < 0.01; as shown in Table 4). Additionally, we found linear correlations between connectivity strength within certain clusters identified by dICA and cognitive scale scores.

**Table 4:** Temporal correlations of FC at the level of seven brain networks.

**Network**	**Frequency**	**Variability**	***p***
SN	√	/	< 0.05
SM	√	/	0.07
LN	/	√	0.07
FPN	/	√	< 0.001
VIN	/	√	< 0.001
SM	/	√	< 0.001
DAN	/	√	< 0.001

This table lists the seven brain networks (SN, SM, LN, FPN, VIN, SM, and DAN), including temporal variability and frequency. Each row represents the statistical values of a brain network, reflecting the variations in these features across different circuits.

In addition, we also to all ICs in temporal frequency and variability analysis, compared to the HC group, the ACS group showed significant differences in the time frequency analysis in the LN (IC_02), and SN (IC_13) (as shown in Table 5); while significant differences in the temporal-variability LN (IC_02), SM (IC_05), SN (IC_10, IC_13), FPN_L (IC_12), and DAN (IC_17) (as shown in Table 6).

**Table 5:** Temporal Frequency correlations at the three ICs.

**ICs**	**Beta**	**T**	***p***	***P* (two-sided)**	**Network**
IC_02	0.0041	2.55	0.007256	0.014512	LN
IC_03	0.0028	2.27	0.014123	0.028246	NA
IC_13	0.0047	2.47	0.008786	0.017572	SN

This table lists the statistical parameters of the temporal frequency correlation of two brain networks, LN (IC_02) and SN (IC_13), and IC_03. Which including Beta, T, *p*, and *p* (two-tailed). Each row represents a statistic for the ICs temporal frequency correlation.

**Table 6:** Temporal Variability correlations at the seven ICs.

**ICs**	**Beta**	**T**	***p***	***P* (two-sided)**	
IC_02	−0.034	−2.52	0.992181	0.015637	LN
IC_03	−0.031	−2.95	0.997380	0.005241	NA
IC_05	−0.058	−5.19	0.999997	0.000006	SM
IC_10	−0.019	−2.05	0.976838	0.046324	SN
IC_12	−0.045	−3.15	0.998487	0.003027	FPN_L
IC_13	−0.049	−3.74	0.999718	0.000563	SN
IC_17	0.052	2.46	0.009025	0.018050	DAN

This table lists the statistical parameters of the temporal variability correlation of six brain networks, LN (IC_02), SN (IC_10, IC_13), SM (IC_05), FPN_L (IC_12) and DAN (IC_17), and IC_03. Which including Beta, T, *p*, and *p* (two-tailed). Each row represents a statistic for the ICs temporal variability correlation.

Finally, the dICA results revealed significant differences in dFC between ACS and HC groups in specific brain regions [F_(2, 27)_ = 10.13, *p*-_uncorrected_ = 0.0005, *p*-FDR = 0.014] (as shown in Figure 3). The connectivity cluster involved the following brain networks: FPN (right dorsolateral prefrontal cortex), SN (bilateral cingulate cortex and left anterior insula), SM (bilateral lateral regions), DAN (right EFE), and CN (bilateral anterior regions). In these dICA results, the ACS group showed enhanced connectivity between the DAN and FPN, CN, and SN, whereas the HC group showed enhanced connectivity between the FPN and CN, and between the SM and SN.

### 3.4 Association analysis

In particular, LN (IC_02) in the ACS group was positively correlated with MoCA (*p* < 0.05), which was negative correlation with MMSE (*p* < 0.05), and delayed recall (*p* < 0.001). Regarding temporal-frequency characteristics, LN (IC_02) in the ACS group was positively correlated with language fluency (*p* < 0.05). In terms of temporal-variability characteristics, FPN_L (IC_06) in the ACS group was positively correlated with digit symbol substitution (*p* < 0.05), while SM (IC_05) was positively correlated with immediate recall (*p* < 0.05) (as shown in Figure 3).

## 4 Discussion

We explored the effective alterations in FC within the brain networks of individuals with unilateral moderate-to-severe ACS compared to those without detected carotid stenosis. ICA highlighted the intrinsic sFC differences in ACS. Through ICA, we identified FC abnormalities in the FPN, DMN, SN, DAN, LN, SM, and CN as well as VIN in individuals with ACS. Among these, FPN, DMN, and SN are the three most important brain networks related to cognitive function. FPN and DMN are involved in cognitive control and decision-making processes, while SN plays a role in a wide range of cognitive control tasks (Cai et al., 2021). Given the complexity and dynamic nature of brain networks, we further performed dICA based on ICA. The results showed mixed intergroup dFC connectivity in the FPN, DMN, SN, DAN, LN, and SM networks in individuals with ACS. Finally, we also assessed the temporal frequency and variability of these six networks. We found that ACS showed differences in the temporal frequency of SN, while significant statistical differences were observed in the temporal variability of LN, FPN, DAN, VIN, and SM.

sFC primarily explores the systematic changes in brain connectivity throughout the lifespan (Deery et al., 2023), mapping and summarizing large-scale functional network patterns across the brain (Zhang et al., 2021; Wang et al., 2021). By resting state fMRI (rs - fMRI) can help us to find the ACS patients brain sFC change and the correlation between cognitive dysfunction. In our study, ICA analysis revealed abnormal connectivity changes at multiple network levels in ACS, particularly with significantly decreased connectivity in brain networks like the LN (IC_02), FPN_L (IC_06, IC_12), and SM (IC_05), while increased connectivity in SN (IC_10) (as shown in Figure 1). Consistent with previous studies, compared to the control group, ACS had disrupted and more asymmetric networks of the DAN, FPN, SMN, and DMN. Specifically, the contralateral insula and dlPFC in the DAN, the contralateral MFG and bilateral IPL in the FPN, the contralateral primary somatosensory cortex, the contralateral supplementary motor cortex in the SMN and the ipsilateral medial prefrontal cortex in the DMN (Lin et al., 2014, 2016). ACS is associated with FC abnormalities across DAN and SN (Gao et al., 2019; Chang et al., 2016). This aligns with the observed impairments in ACS patients across various cognitive domains, such as language memory, working memory, executive function, and perception-related tasks (Norling et al., 2019; Huang et al., 2020; Gao et al., 2021).

However, sFC lacks specificity. At the whole-brain level, sFC can reveal differences in intrinsic networks that are generally present across different populations (Spronk et al., 2021), but the spatial heterogeneity of intrinsic networks in patients with various neuropsychiatric disorders and healthy individuals is relatively small (Zhang et al., 2021; Zhao B. et al., 2022). For example, comorbidities of anxiety and depression involve FPN, DMN (Li et al., 2020; Zhang et al., 2023). AD and Parkinson's disease share cognitive dysfunction-related regions included cingulate and high frontoparietal cortices (Choi et al., 2022). As another example, index negative self-related rumination with DMN hyperconnectivity, which has been observed in people with depression or at high risk (Whitfield-Gabrieli et al., 2020), has also been shown impaired large-scale networks including FPN, DAN, and ventral attention network are related to attention deficits, both in schizophrenia and major depressive disorder patients (Li et al., 2024). In addition, the resting state FC is based on the assumption that FC is time-static throughout the measurement period, and the actual brain activity is highly dynamic and conditional dependent. In spite of sFC can reveal the stability and strength of connectivity within the brain network at a specific time point, and find the relationship between ACS cognitive impairment and brain dysfunction, it cannot capture the fluctuating and time-varying nature of brain activity, and lacks the understanding of the dynamic changes of brain connectivity.

In contrast, dFC identifies variations in brain activity over different time points, capturing more detailed neural network fluctuations and temporal variability (Preti et al., 2017). dFC can not only provide time-varying information of FC between static connectivity networks, but also capture reproducible connectivity states and calculate temporal attributes (Calhoun et al., 2014), that is, dFC can evaluate FC changes in a short period of time, which makes it possible to study the different connection patterns that repeat over a short period of time in brain networks and the fluctuations in their interactions. dFC emphasizes the temporal differences in FC patterns of brain intrinsic networks among patients with different diseases, aiming to explore disease-specific connectivity patterns. By considering time fluctuations within different windows to calculate time-varying FC, and selecting different dFC states to quantify the stability and variability of brain dynamics (Hindriks et al., 2016). As dFC analysis can extract more time-varying characteristics of information exchange between brain regions on a time scale and because these characteristics are significantly related to many physiological parameters (Zhu et al., 2021), pathological features (Zhu et al., 2020), this approach can help detect subtler network changes, even in preclinical stages (Xue et al., 2021). These dynamic changes reflect the brain's short-term adaptive adjustments, transient dysfunctions, or compensatory mechanisms, offering deeper clinical insights compared to sFC. Therefore, dFC may provide significant advantages in understanding brain dysfunctions associated with ACS. For instance, studies by Hindriks et al. (2016) and Sang et al. (2023) demonstrate that dFC can capture network connectivity state fluctuations that sFC might overlook. This capability makes dFC an effective analytical approach for investigating brain functional fluctuations and compensatory mechanisms in clinical conditions such as ACS. Our results indicate mixed patterns of connectivity enhancement and reduction in cognitive networks among ACS patients, we further applied dICA to investigate dFC abnormalities at the network level in ACS patients. Our results revealed intergroup differences in the dFC of six network regions: the DAN, LN, FPN, DMN, and SM, SN, and differed significantly in the FC of LN, FPN, DAN, VIN, and SM in time-variability (*p* < 0.01). For example, AD or amnestic mild cognitive impairment have reduced dFC in the DMN and DAN (Zhao C. et al., 2022). Major depressive disorder patients have reduced dFC in the DMN and executive network (Zhu et al., 2020).

The FPN is tightly connected with other parts of the brain and serves as a crucial hub in cognitive processes (Schmahmann, 2019). The dlPFC, a core component of the FPN, plays a vital role in executive functions, helping to coordinate and integrate the functions of other brain regions. It is involved in working memory, verbal execution, and other cognitive tasks (Panikratova et al., 2020). Abnormal activation of the dlPFC is typically associated with cognitive impairments. Additionally, the Cerebellum_crus1 is involved in cognitive processes related to language memory (Stoodley and Schmahmann, 2009). Previous research has demonstrated reduced FC in the dlPFC (Harding et al., 2015; Avirame et al., 2015) and MFG (Tuo et al., 2021) in ACS, consistent with our results. This study showed that ACS is associated with abnormal connectivity in the FPN, with ICs differences involving brain regions such as the frontal, temporal, and cerebellar areas. Specifically, reduced FC in the dlPFC.R, MFG.R, FFG,R, ITG.R, and bilateral cerebellum superior crus I may explain the poorer overall cognition, psychomotor speed/executive function, memory function, and daily living abilities observed in ACS patients (Gao et al., 2021; Wang et al., 2017). In clinical settings, many ACS patients exhibit normal or only mild cognitive impairments. This may be due to compensatory mechanisms, such as increased dFC in regions including the right MFG, INS.R, ORBinf.R, SFG.L, IPL.L, and bilateral MCG and SMG. These enhancements in connectivity may help maintain relatively better cognitive performance in certain domains for ACS patients (Jia et al., 2014; Schoonheim et al., 2022).

The functional interaction between the FPN and the DMN is crucial for the expression of executive functions and working memory. DMN plays a vital role in the normal activation and maturation of FPN (Chen et al., 2023). In many neuropsychiatric disorders that lead to cognitive impairment, DMN is often the first affected brain network (Zhang and Raichle, 2010). In our study, we observed that in ACS, the dFC of the SMA.R in the DMN was reduced, while the dFC of the PCUN.R and FFG.L was increased. Both increased and decreased FC are closely related to cognitive dysfunction. The SMA not only plays a role in motor-related functions but also participates in higher-order cognitive control mechanisms, coordinating language fluency, and attention switching (Hertrich et al., 2016). The PCUN is a central node of the DMN, and its FC abnormalities are associated with impairments in cognitive abilities such as episodic memory and working memory (Cavanna and Trimble, 2006). Current research suggests that ACS patients typically exhibit reduced FC in the PCUN and SMA (Maimaitiaili et al., 2024; He et al., 2020), which differs from our findings. We found increased dFC in the PCUN.R. Some studies suggest that increased PCUN dFC may serve as a protective factor for cognitive functions related to memory (Zhao C. et al., 2022). Based on this, we hypothesize that the heightened dFC in the PCUN and FFG in ACS may represent a compensatory mechanism, where enhanced connectivity helps maintain normal memory consolidation, working memory, and the interaction between emotional processing and cognitive function.

Our study found that in ACS patients, the reduced dFC in the SMA may contribute to language impairments. Additionally, the reduced dFC in the STG.L and MTG.L is noteworthy. The temporal pole, as one of the auxiliary language areas outside of Wernicke's area, supports the retrieval of phonological information, which is critical for speech output and short-term memory tasks. This reduction in connectivity is associated with impaired cognitive performance in speech output and short-term memory tasks (He et al., 2020). Moreover, the traditional language areas largely overlap with left-lateralized co-activation regions of the brain, which are strongly correlated with the execution of language tasks. Left hemispheric lateralization is significantly positively associated with impaired language task performance (Peng et al., 2023). Therefore, the reduced dFC in the lateralized regions such as the STG.L, MTG.L, and SMA.R may be a potential mechanism underlying the poorer language and memory performance in ACS patients.

In this study, we found that ACS patients exhibited reduced dFC in the PreCG.L and SPG.R within the DAN. The SPG is involved in top-down attentional orientation, and its dysfunction is closely associated with memory heterogeneity (Koenigs et al., 2009). FC analysis, by revealing the dynamic changes in brain networks over time, offers a unique perspective through dFC. In this study, we observed significant changes in the temporal frequency and variability within networks such as the SN, LN, and FPN in ACS patients. These changes may reflect adaptive adjustments under cognitive load or environmental stress. For example, the temporal frequency changes in the SN suggest dynamic adaptation in the allocation of cognitive resources when facing external stimuli, while the temporal variability in the LN and DMN may be related to dynamic changes in memory and language functions. These findings indicate that the transient connectivity patterns captured by dFC could serve as biomarkers for early cognitive impairment, offering new insights for diagnosis and intervention. Furthermore, dFC also revealed compensatory adjustments in the DAN and SM in ACS patients. The temporal variability changes in these networks may reflect transient adaptive mechanisms in attention allocation and motor control, which are difficult to detect through sFC analysis. Thus, the dFC findings in this study not only expand our understanding of brain functional changes in ACS but also suggest that dFC may have clinical value in the early identification of potential cognitive decline. Previous research on MCI has shown that the SPG mediates processes such as short-term memory, delayed recall, and memory recognition (Zhong et al., 2022). These findings provide valuable insight into the relationship between the SPG and ACS, suggesting that the SPG may serve as a potential target for neuroregulation in ACS patients.

The CN, often overlooked, includes the cerebellum, which not only plays a role in motor control but is also crucial for coordinating emotional and visceral functions, making sensory predictions, and engaging in higher cognitive functions such as reasoning (Gao et al., 1996). Damage to the cerebellum has been associated with deficits in emotional attribution and social skills (Schmahmann, 2019). In this study, we found increased connectivity within the CN in ACS patients, which may underlie the neural mechanisms leading to impaired perception of bodily, functions, balance during walking, and difficulties in daily activities (Gray et al., 2020).

Most notably, our study captured the temporal characteristics of FC and revealed time-related FC abnormalities in ACS. Specifically, the SN showed differences in time-frequency FC, while the LN, FPN, DAN, VIN, and SM exhibited significant differences in time variability of FC. These findings suggest that such temporal FC abnormalities may be potential mechanisms underlying the impairments in various aspects of cognitive and motor control in ACS patients.

## 5 Limitations

Although this study identified dFC abnormalities and dynamic temporal variability across multiple networks in ACS patients, shedding light on potential mechanisms underlying cognitive impairment, several limitations remain. First, this study is a derivation study and does not include a validation dataset, which limits the generalizability of the findings. We plan to include independent datasets in future research to enhance the robustness of the results. Second, the sample size is relatively small, which may affect the applicability of the conclusions to larger populations. Third, we did not perform group comparisons between patients with left- and right-sided stenosis, which may have underestimated the effect of laterality on brain function. Fourth, this study focused solely on patients with unilateral moderate-to-severe stenosis who had not undergone surgical intervention. As a result, the findings may not apply to patients with restenosis after carotid stenting or those with mild or bilateral stenosis. Lastly, this study employed a cross-sectional design, lacking longitudinal data to evaluate changes in FC over time. Future studies should incorporate longitudinal data and further investigate the relationship between imaging metrics and cognitive function.

## 6 Conclusions

This study demonstrates that cognitive impairment in ACS is strongly associated with significant alterations in both intrinsic sFC and dFC within large-scale brain networks. dICA revealed temporal frequency changes in the SN, further suggesting that ACS may influence the temporal characteristics of brain connectivity. Additionally, significant temporal variability in FC was observed in several key networks, including the FPN, DAN, SMN, LN, and VIN. These dynamic changes were closely related to cognitive decline, particularly in language, memory and executive function. Our findings emphasize the importance of considering both static and dynamic connectivity changes in brain network when assessing the cognitive impacts of ACS. The results indicate that ACS not only disrupts large-scale brain network connectivity but also impairs the temporal dynamics of brain network interactions, which may contribute to the cognitive and motor deficits observed in ACS patients.

## Data Availability

The raw data supporting the conclusions of this article will be made available by the authors, without undue reservation.
